# How Immunotherapy Has Redefined the Treatment Paradigm of Metastatic or Locally Advanced Muscle-Invasive Urothelial Bladder Carcinoma

**DOI:** 10.3390/cancers16091780

**Published:** 2024-05-05

**Authors:** Mathieu Larroquette, Félix Lefort, Charlotte Domblides, Luc Héraudet, Grégoire Robert, Alain Ravaud, Marine Gross-Goupil

**Affiliations:** 1 Medical Oncology Department, Hôpital Saint André, University Hospital of Bordeaux, 33076 Bordeaux, France; 2 Bordeaux University, CNRS UMR 5095, IBGC, 33076 Bordeaux, France; 3 ImmunoConcEpt, CNRS UMR 5164, Bordeaux University, 33076 Bordeaux, France; 4 Urology Department, University Hospital of Bordeaux, 33076 Bordeaux, France

**Keywords:** metastatic urothelial carcinoma, immune checkpoint inhibitor, enfortumab vedotin, ESMO guidelines

## Abstract

**Simple Summary:**

Metastatic bladder carcinoma is a cancer with a poor prognosis, for which treatments have remained the same for several decades. Immunotherapy has revolutionized the management of several solid cancers, significantly improving the prognosis of some patients. Several protocols have been evaluated this treatment in bladder cancer, in first or second line, with conflicting results. However, two recent studies evaluating immunotherapy either with cisplatin-based chemotherapy or with enfortumab vedotin, an antibody-drug conjugated (ADC), have demonstrated their benefits in terms of overall survival as a first-line treatment, thus redefining the standard of care for patients. Immunotherapy is also being evaluated in the peri-operative setting, with encouraging results for patients with localized or locally advanced bladder cancer.

**Abstract:**

In the past decade, the therapeutic arsenal for metastatic bladder cancer has expanded considerably, with the development of immune checkpoint inhibitors (ICIs), antibody–drug conjugates such as enfortumab vedotin, and anti-fibroblast growth factor receptor agents. Clinical trials evaluating ICIs as neoadjuvants, adjuvants, or first- or second-line treatments have produced conflicting results. However, first-line therapeutic strategies have been redefined by the recent publication of results from two clinical trials: CheckMate-901, which demonstrated the superiority of combined treatment with nivolumab and chemotherapy in extending overall survival, and EV-302, which demonstrated that combined treatment with pembrolizumab and enfortumab vedotin reduced the risk of death by 53%. In this review, we discuss the role of ICIs, alone or in combination, in bladder cancer management in the metastatic and adjuvant settings in 2024, considering the latest published trials. The potential role of ICIs as neoadjuvants is also discussed.

## 1. Introduction

Bladder cancer has an annual incidence of more than 550,000 new cases and is responsible of more than 200,000 deaths worldwide each year, with the highest incidence occurring in Europe and North America [1]. The current standard of care for patients with non-metastatic muscle-invasive urothelial carcinoma (UC) consists of surgery, following neoadjuvant cisplatin-based chemotherapy for eligible patients [2]. For several decades, first-line treatment of locally advanced or metastatic UC has relied on cisplatin-based chemotherapy, with a median overall survival (OS) of 14 months. However, up to 50% of patients are considered ineligible for cisplatin and receive, instead, combined carboplatine and gemcitabine [3,4,5]. The therapeutic arsenal for progression after first-line therapy has improved considerably in recent years. Patients with alterations in fibroblast growth factor receptor (FGFR) 2 or 3 can benefit from erdafitinib, an FGFR1–4 inhibitor that offers an OS advantage over chemotherapy [6]. Enfortumab vedotin (EV), an antibody–drug conjugate (ADC) directed against Nectin-4, a cell surface antigen expressed in the majority of UC cases, has also provided an OS advantage over chemotherapy in pretreated patients. Among other innovative treatments, immune checkpoint inhibitors (ICIs), which mainly target the programmed cell death protein 1 (PD-1)/programmed death-ligand 1 (PD-L1) axis, have been widely evaluated in UC. Thus, avelumab is indicated for maintenance therapy after platinum plus gemcitabine in the first-line setting, pembrolizumab is recommended for pretreated patients, and both pembrolizumab and atezolizumab have been approved as first-line alternatives for cisplatin-ineligible patients, with authorisation dependent on PD-L1 expression [2]. Recently, ICIs have been evaluated in the front-line setting in combination with chemotherapy or EV, leading to major changes in patient outcomes. Furthermore, immunotherapy in the adjuvant setting has been approved for selected patients after surgery. In this review, we discuss the results of trials that have evaluated ICIs in the first- and second-line settings, highlighting the main advancements in terms of clinical practice. We also discuss current evaluations of ICIs in the neoadjuvant and adjuvant settings, where they have shown promising results.

## 2. ICIs for Platinum-Pretreated Patients with Advanced UC

Although standard first-line therapies for advanced UC have been strongly based on cisplatin and gemcitabine for several decades, second-line treatment options remained an unmet need, with no standard recommended protocol. In this context, ICIs were first evaluated in the second-line setting, leading to therapeutic improvements in this area prior to upfront evaluation.

In the KEYNOTE-045 randomised phase 3 trial, pembrolizumab was administered versus the investigator’s choice of chemotherapy (paclitaxel, docetaxel, or vinflunine) in patients who had experienced recurrence or progression after platinum-based chemotherapy. The study included 542 patients. After a median follow-up of 14.1 months, the results showed a significant OS benefit with pembrolizumab (median OS: 10.3 months vs. 7.4 months, hazard ratio [HR]: 0.73, 95% confidence interval [CI]: 0.58–0.91 months; *p* = 0.002), leading to its approval by the US Food and Drug Administration (FDA) for this indication (Table 1). The median progression-free survival (PFS), which was the co-primary endpoint of the study with OS, was not significantly different between the two arms (2.1 months vs. 3.3 months, HR: 0.98, 95% CI: 0.81–1.19 months; *p* = 0.42). However, after 6–8 months of treatment, the PFS curve began to reach a plateau in the pembrolizumab arm, with a greater 12-month PFS than in the chemotherapy arm (16.8% vs. 6.2%) [7]. These results were confirmed in an updated follow-up of the trial, which reported a 48-month OS of 16.7% vs. 10.1% and a 48-month PFS of 9.5% vs. 2.7% in favour of pembrolizumab [8].

Atezolizumab was first evaluated in a single-arm, two-cohort phase 2 trial including 310 patients with advanced or metastatic UC progressing after platinum-based chemotherapy. The study showed a 15% objective response rate (ORR) in the intention-to-treat (ITT) population, and 27% in the IC2/3 subgroup (≥5% of tumour-infiltrating immune cells), leading to the accelerated FDA approval of atezolizumab for metastatic UC [9] (Table 1). However, the IMvigor211 phase 3 trial did not confirm these results, with negative OS and PFS in the IC2/3 subgroup, leading to the removal of FDA approval of atezolizumab for this indication [10].

**Table 1:** Clinical trials evaluating immune checkpoint inhibitors (ICIs) in advanced or metastatic urothelial carcinoma.

Trial (Year of 1st Publication)	Phase	Treatment	OS	ORR	FDA or EMA Approval *
**Evaluation of ICI combination therapies in first-line treatment**					
EV-302 (2024) [11]	III	Enfortumab–vedotin + pembrolizumab vs. platinum–gemcitabine	Median OS: 31.5 vs. 16.1 mo HR: 0.46 95% CI: 0.38–0.58 (*p* < 0.00001)	67.7% vs. 44.4%	FDA
Checkmate-901 (2023) [12]	III	Nivolumab/placebo + cisplatin–gemcitabine	Median OS: 21.7 vs. 18.9 mo HR: 0.78 95% CI: 0.63–0.96 (*p* = 0.02)	57.6% vs. 43.1%	FDA
IMvigor130 (arm A vs. arm C) (2020) [13]	III	Atezolizumab/placebo + platinum–gemcitabine	Median OS: 16.1 vs. 13.4 mo NS	48.1% vs. 44.8%	Not approved
KEYNOTE-361 (2021) [14]	III	Pembrolizumab/placebo + platinum–gemcitabine	Median OS: 17 vs. 14.3 mo NS	54.7% vs. 44.9%	Not approved
GCISAVE (2024) [15]	II	Avelumab + cisplatin–gemcitabine vs. cisplatin–gemcitabine	Prematurely stopped	79.5% vs. 59.1%	Not approved
**ICI maintenance after no progression under first-line platinum-based chemotherapy**					
JAVELIN Bladder 100 (2020) [16]	III	Avelumab + BSC vs. BSC	Median OS: 23.8 vs. 15.0 mo HR: 0.76 95% CI: 0.63–0.91 (*p* = 0.0036)	–	FDA and EMA
**Evaluation of first-line ICIs as monotherapy**					
KEYNOTE-361 (2021) [14]	III	Pembrolizumab vs. platinum–gemcitabine	Median OS: 15.6 vs. 14.3 mo NS	30.3% vs. 44.9%	FDA and EMA (Cisplatin-ineligible patients only; cf. KEYNOTE-052)
IMvigor130 (arm B vs. arm C) (2020) [13]	III	Atezolizumab vs. platinum–gemcitabine	Median OS: 15.2 vs. 13.3 mo NS	23% vs. 44%	EMA (Cisplatin-ineligible patients only; cf. IMvigor-210)
DANUBE (2020) [17]	III	Durvalumab + tremelimumab vs. platinum–gemcitabine Durvalumab vs. platinum–gemcitabine	Median OS: 15.1 vs. 12.1 mo NS Median OS: 14.4 vs. 12.1 mo (PD-L1 ≥ 25% population)	36% vs. 49% 28% vs. 48% (PD-L1 ≥ 25% population)	Not approved
IMvigor210 (2017) [18]	II	Atezolizumab in cisplatin-ineligible patients	Median OS: 15.9 mo	23%	EMA (for tumours with PD-L1 ≥ 5%)
KEYNOTE-052 (2017) [19]	II	Pembrolizumab in cisplatin-ineligible patients	Median OS: 11.3 mo (ITT population) Median OS: 18.5 mo (CPS ≥ 10 population)	28.9% (ITT population) 47.3% (CPS ≥ 10 population)	FDA (for patients not eligible for any platinum-containing chemotherapy) EMA (CPS ≥ 10 population)
**Evaluation of ICIs in the second-line setting**					
KEYNOTE-045 (2017) [7]	III	Pembrolizumab vs. chemotherapy	Median OS: 10.3 vs. 7.4 mo HR: 0.73 95% CI: 0.58–0.91 (*p* = 0.002)	21.1% vs. 11%	FDA and EMA
IMvigor211 (2017) [10]	III	Atezolizumab vs. chemotherapy	Median OS: 11.1 vs. 10.6 mo NS (IC2/3 population)	23% vs. 22% (IC2/3 population)	EMA (for tumours with PD-L1 ≥ 5%)
Checkmate-275 (2017) [20]	II	Nivolumab	Median OS: 8.6 mo	20.7%	FDA and EMA

BSC, best supportive care; CI, confidence interval; CPS, combined positive score; EMA, European Medicines Agency; FDA, United States Food and Drug Administration; HR, hazard ratio; IC, tumour-infiltrating immune cells; ITT, intention-to-treat; mo, months; NS = not significant; ORR, objective response rate; OS, overall survival; PD-L1, programmed death-ligand 1. * Status on 22 March 2024.

Nivolumab obtained accelerated FDA approval for pretreated advanced UC, based on the results of the CheckMate 275 single-arm phase II trial, which included 270 patients receiving nivolumab after platinum-based chemotherapy given in the metastatic (66%) or neoadjuvant/adjuvant (34%) setting. The first results to be published from this study reported an ORR of 19.6%, including an ORR of 16.1% in patients with <1% PD-L1 expression. Extended follow-up of this cohort confirmed an ORR of 20.7% and median OS of 8.6 months for the ITT population [20] (Table 1). Interestingly, an exploratory analysis showed an association between ORR and higher tumour mutational burden in evaluable patients [21].

To date, avelumab and durvalumab have been evaluated only in platinum-pretreated patients with advanced or metastatic UC in early phase trials, which have reported ORRs of 17% and 17.8%, respectively [22,23]. These results led to accelerated FDA approval of both products for this indication; however, approval for durvalumab was withdrawn in 2021, following the negative results of the DANUBE phase III trial in the first-line setting [24].

Pembrolizumab is the only ICI that has shown an OS improvement in a large phase III randomised controlled trial (RCT) and, therefore, should be the preferred treatment in second-line immunotherapy for metastatic UC patients progressing after a platinum regimen in the first line [25].

## 3. ICIs as First-Line Therapy for Advanced or Metastatic UC

### 3.1. ICIs as Single Agents

#### 3.1.1. Evaluation Irrespective of Cisplatin Eligibility

Three phase III RCTs have evaluated ICI monotherapy in the first-line setting compared with platinum-based chemotherapy. Among the three arms of the KEYNOTE-361 trial, one arm evaluated treatment with pembrolizumab alone versus treatment in combination with gemcitabine. Comparison analyses between these two arms were exploratory and could not be formally tested due to the design of the study; however, they provided interesting results. OS analysis showed no difference between the two groups, either in the ITT population (median: 15.6 months vs. 14.3 months, HR: 0.92, 95% CI: 0.77–1.11 months) or in the combined positive score (CPS) > 10 population (median OS: 16.2 months vs. 15.2 months, HR: 1.01, 95% CI: 0.77–1.32) (Table 1). Notably, the ORR for the pembrolizumab group was only 30.3% compared with 44.9% in the chemotherapy group, and OS was numerically higher in the chemotherapy group than in the pembrolizumab group for the first 12 months of the study, suggesting a potential deleterious effect of pembrolizumab alone [14].

Atezolizumab monotherapy has also been evaluated in comparison with combined therapy, with gemcitabine and platinum, in group B of the IMvigor130 phase III trial. Due to the negative results of the first part of the study, the OS analysis of atezolizumab monotherapy versus chemotherapy could not be formally tested and should be considered exploratory [13]. Nevertheless, the recently published final OS analysis showed no superiority for atezolizumab over chemotherapy (median OS: 15.2 months vs. 13.3 months, HR: 0.98, 95% CI: 0.82–1.16), with a numerically lower OS rate in patients receiving atezolizumab for the first 9 months of treatment. Interestingly, other exploratory analyses have shown that a subset of patients with high PD-L1 expression (IC2/3) tend to have longer OS with atezolizumab (median OS: 27.5 months vs. 16.7 months, HR: 0.70, 95% CI: 0.48–1.03) [26] (Table 1).

The latest phase III RCT was the DANUBE study, in which patients received either durvalumab monotherapy or durvalumab–tremelimumab combination therapy against gemcitabine plus platinum chemotherapy. This study showed negative results for OS, its co-primary endpoint, with no advantage for either durvalumab monotherapy in the PD-L1 > 25% population or the durvalumab–tremelimumab combined therapy in the ITT population, versus chemotherapy. Notably, secondary analyses have shown a median OS of 17.9 months with durvalumab–tremelimumab in the high-PD-L1 population, which was better than the one observed with chemotherapy (HR: 0.74, 95% CI: 0.59–0.93) (Table 1). Durvalumab, as either mono- or bi-therapy, also led to lower OS in the initial months of treatment, associated with lower ORR compared with chemotherapy, highlighting a potential loss of opportunity for non-responders [17].

#### 3.1.2. Evaluation in Cisplatin-Ineligible Patients

Cisplatin ineligibility affects up to 50% of advanced UC patients, mainly due to impaired renal function (glomerular filtration rate [GFR] < 50–60 mL/min), Eastern Cooperative Oncology Group status ≥ 2, grade ≥ 2 hearing loss, peripheral neuropathy, or New York Heart Association class III heart failure [3]. Standard treatment for these patients has relied on carboplatine-based combination chemotherapy, which is less efficient than cisplatin-based regimens, with shorter OS and lower ORR [27,28]. Therefore, ICIs have been evaluated for this specific population in the hope of improving treatment efficiency.

IMvigor210 and KEYNOTE-052, two single-arm phase II trials for the first-line treatment of patients with metastatic UC who were ineligible for cisplatin showed ORRs of 23% and 24% with atezolizumab and pembrolizumab, respectively. In the IMvigor210 study, the median OS was 15.9 months for patients receiving atezolizumab [18,19] (Table 1). The results of these trials led to temporarily accelerated FDA approval for these two ICIs in the first-line setting for platinum-ineligible patients.

Exploratory analysis results from phase III trials have also produced results for the cisplatin-ineligible population. In the KEYNOTE-361 trial, OS results for the 56% of control patients who received carboplatine did not show a significant advantage of pembrolizumab over chemotherapy in both the CPS > 10 and ITT populations [14]. In the IC2/3 subset of patients in the IMvigor130 trial, pembrolizumab provided longer OS in the cisplatin-ineligible population (median OS: 18.6 months vs. 10.0 months, HR: 0.56, 95% CI: 0.34–0.91); however, these data represent a small number of patients (N = 93) and should be interpreted with caution, as the study was not designed to address this research question [13,26]. In the DANUBE trial, ORRs were rather similar between cisplatin-eligible and cisplatin-ineligible patients, in both the chemotherapy and ICI arms [17]. Again, these trials were not designed to address these specific comparisons, and the results should be considered exploratory.

Regarding the negative results of the IMvigor130 and DANUBE trials with respect to their primary endpoints, FDA approval of atezolizumab and durvalumab for UC was withdrawn. To date, pembrolizumab remains the only ICI that is FDA approved as a first-line treatment option for patients who are ineligible for any platinum-containing chemotherapy.

### 3.2. ICI Maintenance after First-Line Platinum-Based Chemotherapy as a Sequential Strategy

In 2020, the first-line standard of care for advanced or metastatic UC underwent a transformation with the introduction of avelumab as maintenance therapy in non-progressive patients after 4–6 cycles of platinum-based chemotherapy. This modification occurred after the results of the phase III RCT Javelin Bladder 100 trial were released. This trial included 700 patients who had experienced no progression after the completion of 4–6 cycles of combined gemcitabine and platinum chemotherapy; they received either avelumab plus best supportive care (BSC) or BSC alone until progression. The study met its primary endpoint, with a median OS of 21.4 vs. 14.3 months in the overall population in the first analysis (HR: 0.69, 95% CI: 0.56–0.86; *p* < 0.001). These results were confirmed after a ≥ 2-year follow-up, with an actualised median OS of 23.8 vs. 15.0 months (HR: 0.76, 95% CI: 0.63–0.91; *p* = 0.0036), and 53% of patients in the BSC group receiving subsequent ICI therapy after progression. The OS benefit was observed both in the PD-L1-positive (PD-L1 ≥ 25% of tumour or immune cells) and PD-L1-negative subgroups (Table 1). Median PFS was also longer with avelumab maintenance in the overall population (5.5 vs. 2.1 months, HR: 0.54, 95% CI: 0.46–0.64; *p* < 0.0001) [16,29]. Notably, exploratory analyses have found a consistent OS benefit in both the subgroup of 269 patients who received first-line combined carboplatin and gemcitabine (HR: 0.69, 95% CI: 0.516–0.925) and in the subgroup of 389 patients who received combined cisplatin and gemcitabine (HR: 0.79, 95% CI: 0.611–1.020) [30,31].

Several non-interventional studies have subsequently evaluated the real-world clinical efficacy of avelumab maintenance. The initial results of the AVENANCE trial, which was conducted in France, were reported in 2023. In this trial, 594 patients received avelumab for advanced or metastatic UC, among whom 61% were treated with first-line carboplatine and gemcitabine. The latest updated analysis of this trial showed a median PFS of 5.7 months and median OS of 21.1 months from the start of avelumab administration [32,33]. Two other observational studies are ongoing, in the USA (PATRIOT II) and Germany (AVENUE), which will provide more real-world data on the efficacy of avelumab maintenance [31,34]. Thus, even if avelumab has become the new standard of care in first-line maintenance, it offers a benefit only to patients who are sensitive to platinum-based chemotherapy.

### 3.3. Combination Strategies with ICI in the First-Line Setting

#### 3.3.1. ICI–Chemotherapy Combination

In the field of first-line combination ICI and chemotherapy, the first positive and most recently published phase III trial was Checkmate-901 [12]. The first part of this trial was conducted to assess the efficacy of adding nivolumab to combined cisplatin–gemcitabine therapy. Only patients eligible for cisplatin were included in this trial. After a median follow-up of 33.6 months, the study showed advantages in terms of both OS (median OS: 21.7 months vs. 18.9 months, HR: 0.78, 95% CI: 0.63–0.96, *p* = 0.02) and PFS (median PFS: 7.9 vs. 7.6 months, HR: 0.72, 95% CI: 0.59–0.88, *p* = 0.001) in the nivolumab plus chemotherapy group, with long-lasting responses (2-year PFS: 23.5% vs. 9.6%) (Table 1). These results were consistent regardless of PD-L1 status (tumour proportion score ≥ 1% or <1%). ORR was also higher in the nivolumab plus chemotherapy group (57.6% vs. 43.1%), with a higher complete response rate (CR) (21.7 vs. 11.8%).

Two other phase III RCTs have evaluated chemotherapy plus ICI as first-line therapy; both were negative for their primary endpoints. The IMvigor130 trial evaluated atezolizumab plus platinum-based chemotherapy (arm A) versus placebo plus platinum-based chemotherapy (arm C). This study was initially designed to include only cisplatin-ineligible patients; however, after it had started, it was amended to recruit cisplatin-eligible patients. Despite a moderate PFS benefit with the addition of atezolizumab to chemotherapy in the ITT population (median PFS: 8.2 months vs. 6.3 months, HR: 0.82, 95% CI: 0.70–0.96; *p* = 0.007), the trial did not cross the statistical boundary for OS [13,35] (Table 1). ORR was numerically slightly higher with the co-administration of atezolizumab (48.1% in arm A vs. 44.8% in arm C). Due to the initial study design, only 30% and 34% of patients received cisplatin in arms A and C, respectively, which may have contributed to negative results in the event of a greater effect of atezolizumab when co-administered with cisplatin, as suggested in a post hoc analysis [36].

In the open-label KEYNOTE-361 trial, patients received platinum-based chemotherapy plus pembrolizumab or placebo [14]. The protocol was designed to recruit both cisplatin-eligible and cisplatin-ineligible patients; a total of 312 (44%) and 391 (56%) patients received cisplatin or carboplatin-based chemotherapy, respectively. The trial did not meet its co-primary endpoint, with no improvement observed in either PFS or OS (Table 1). However, in the pembrolizumab + chemotherapy arm, patients who received cisplatin tended to have longer PFS (HR: 0.67, 95% CI: 0.51–0.89) than those who received carboplatin (HR: 0.86, 95% CI: 0.68–1.09). Moreover, following the addition of pembrolizumab to chemotherapy, ORR increased from 41.8% to 47.2% and from 48.7% to 64.1% in the carboplatin and cisplatin groups, respectively, suggesting a potential benefit of pembrolizumab when administered with cisplatin.

The efficacy of avelumab was also investigated upfront, in combination with cisplatin and gemcitabine, in the randomised phase II GCISAVE trial, which reported an encouraging ORR of 79.5% versus 59.1% for the addition of avelumab, compared to cisplatin + gemcitabine alone [15]. However, this trial was prematurely stopped following the approval of avelumab maintenance in the first-line setting.

Among these trials, only nivolumab has demonstrated a survival benefit when combined with first-line standard chemotherapy. One possible reason for these differences is the heterogeneity of the population of inclusion. In the Checkmate-901 trial, patients received only cisplatin-based chemotherapy, whereas the majority of patients in the IMvigor130 and KEYNOTE-361 trials received carboplatin. The positive Checkmate-901 results, and the benefit observed in subgroups who received cisplatin in the two other trials, suggest that cisplatin may drive the survival advantage provided by ICIs. This hypothesis is consistent with preclinical data, which demonstrate that cisplatin promotes T cell tumour infiltration and Th1 differentiation, turning “cold” tumours into “hot” tumours.

No new safety signals were reported for nivolumab plus cisplatin and gemcitabine combination therapy; grade ≥ 3 treatment-related adverse events (TRAEs) occurred in 61.8% and 51.7% of patients in the ICI-chemotherapy and chemotherapy groups, respectively. The rate of grade ≥ 3 hematologic disorders was slightly increased in the nivolumab + chemotherapy group (anaemia: 22% vs. 17.7%, neutropenia: 18.8% vs. 15.3%, thrombocytopenia: 6.6% vs. 4.5%, respectively). The rates of diarrhoea and pruritus (any grade) were increased in the nivolumab + chemotherapy combination group (13.2% vs. 8.7% and 14.5% vs. 2.8%, respectively), with only four cases of grade ≥ 3 diarrhoea in the nivolumab + chemotherapy group.

#### 3.3.2. ICI–ADC Combination

Monomethyl auristatin E, the cytotoxic payload component of the ADC EV, is a microtubule-disrupting agent that can also induce immunogenic cell death and activate antigen-presenting cells [37,38,39]. These properties underpin the rationale for studying combined therapy with EV and ICI.

The EV-103 phase Ib/II trial evaluated the combination of EV (administered on days 1 and 8) plus pembrolizumab (day 1) in the first-line setting in 55 cisplatin-ineligible patients. The results of the dose expansion and dose escalation parts of the trial were promising, with an ORR of 73.3% and median OS of 26.1 months [40]. In cohort K of the same trial, patients were randomly assigned to receive either EV plus pembrolizumab or EV monotherapy. The results showed an increased ORR with the combination of the two drugs compared with EV monotherapy (64.5% vs. 45.2%) [41].

These encouraging results led to the EV-302 phase III study, in which cisplatin and carboplatine-eligible patients were randomly assigned to receive either EV plus pembrolizumab or gemcitabin plus carboplatine/cisplatin. The initial results of this trial were presented in a plenary session during the 2023 European Society for Medical Oncology (ESMO) congress and were subsequently published. The study met its dual primary endpoint, showing both a drastically improved PFS (median PFS: 12.5 vs. 6.3 months, HR: 0.45, 95% CI: 0.38–0.54; *p* < 0.00001) and OS (median OS: 31.5 vs. 16.1 months, HR: 0.47, 95% CI: 0.38–0.58; *p* < 0.00001) with EV plus pembrolizumab (Table 1). The experimental combination also provided an ORR of 67.7% versus 44.4% in the chemotherapy arm [11]. Subgroup analyses recently presented at the 2024 American Society of Clinical Oncology Genitourinary (ASCO-GU) Cancer Symposium showed a consistent benefit across subgroups, including patients who were eligible or ineligible for cisplatin, as defined by their renal function (HR: 0.51, 95% CI: 0.30–0.86 vs. HR: 0.44, 95% CI: 0.30–0.65 vs. HR: 0.50, 95% CI: 0.37–0.69 for normal vs. mild vs. moderate/severe renal function, respectively) [42]. Following the dissemination of these results, the EV + pembrolizumab combination obtained FDA approval for first-line treatment of metastatic or locally advanced UC [43].

In the EV-302 trial, the most common TRAEs with increased rates in the EV + pembrolizumab group were peripheral sensory neuropathy (50%), pruritus (39.8%), alopecia (33.2%), and maculopapular rash (32.7%). Grade ≥ 3 TRAEs occurred in 55.9% of patients in the pembrolizumab + EV arm, compared to 69.5% in the chemotherapy arm. The most common grade ≥ 3 TRAEs that were mainly related to EV were skin reactions (15.5%), peripheral neuropathy (6.8%), and hyperglycaemia (6.1%). TRAEs resulted in treatment discontinuation in 35% of patients receiving EV + pembrolizumab (discontinuation of EV in 29.5% of patients and of pembrolizumab in 21.4% of patients) versus in 18.5% of patients receiving chemotherapy.

## 4. ICIs as Adjuvant Therapy

The benefit of cisplatin-based adjuvant chemotherapy after complete resection of muscle invasive bladder cancer (MIBC) in patients with a high risk of recurrence (pT3-T4 and/or N+) remains controversial due to limited RCT-based evidence, although a recent meta-analysis tended to show an OS advantage [44]. Three phase III RCTs were conducted to investigate whether ICI administration in this setting could enhance disease-free survival (DFS) and OS.

The CheckMate 274 trial evaluated 1 year of treatment with nivolumab versus placebo in patients with pT3-4a or pN+ tumours after surgery. The results were positive for both primary endpoints of the study, with a significant DFS improvement following nivolumab therapy in the overall population (median DFS: 20.8 vs. 10.8 months, HR: 0.70, 95% CI: 0.55–0.90; *p* < 0.001) and in patients with PD-L1 expression ≥ 1% (HR: 0.55, 95% CI: 0.35–0.85; *p* < 0.001) [45]. The actualised results of this trial after a median follow-up of 3 years confirmed the primary data for DFS [46]; OS analysis results have not yet been reported.

The IMvigor010 trial compared 1 year of atezolizumab therapy versus observation after radical resection of UC, with ypT2-T4a or ypN+ residual disease for patients who received neoadjuvant chemotherapy or pT3-4a or pN+ for patients without prior chemotherapy and ineligible for adjuvant cisplatin. The trial showed negative results, with no differences in DFS (median DFS: 19.4 vs. 16.6 months, HR: 0.89, 95% CI: 0.74–1.08; *p* = 0.24) or OS (HR: 0.85, 95% CI: 0.66–1.09) with the administration of atezolizumab [47]. An interim exploratory analysis of the trial data found that patients with detectable circulating tumour DNA after surgery were more able to benefit from atezolizumab, with an increased OS (HR: 0.59, 95% CI: 0.42–0.83) [48]. The IMvigor011 trial was designed to evaluate the efficacy of atezolizumab in patients included after surgery and randomised between observation or atezolizumab in case of circulating tumour DNA positivity [49], such that these results are meaningful.

The AMBASSADOR trial evaluated pembrolizumab versus observation in the same population (but also including patients with positive margins after resection). The primary results were presented at the 2024 ASCO-GU congress after a median follow-up of 22.3 months for DFS and 36.9 months for OS, representing the co-primary endpoints of the study. Median DFS was improved (29 vs. 14 months, HR: 0.69, 95% CI: 0.55–0.87; *p* = 0.0013), whereas median OS was similar with pembrolizumab (50.9 vs. 55.8 months, HR: 0.98, 95% CI: 0.76–1.26; *p* = 0.88) [50].

To date, only the CheckMate 274 trial has met its primary endpoint, showing a significant improvement of DFS with nivolumab. With the occurrence of only 257 events in 702 included patients, the OS data of the AMBASSADOR trial are likely to be immature, and its results could turn out to be positive after extended follow-up. Although cross-trial comparison should be conducted with caution, it is worth noting that the DFS for the control arm was slightly shorter in Checkmate 274 than in IMvigor010 (10.8 vs. 16.6 months), whereas the experimental groups had more similar DFS rates (19.4 vs. 20.8 months, respectively), despite a similar inclusion population. Based on these results, nivolumab has been FDA approved for adjuvant treatment of patients who are at high risk of recurrence after radical resection. Approval by the European Medicines Agency is restricted to patients with PD-L1 ≥ 1% for tumour cells. Mature OS data for these trials are eagerly awaited.

## 5. Evaluation of ICIs in the Neoadjuvant Setting

Neoadjuvant chemotherapy is currently recommended only for cisplatin-eligible patients, with the dose-dense methotrexate/vinblastine/doxorubicin/cisplatin regimen providing the best local control rate at a complete pathological response (CPR) rate of 42% obtained in the VESPER trial [51]. There is an unmet need to provide new therapeutic options for cisplatin-ineligible patients and to increase the disease control rate. Due to their efficacy in the metastatic setting, ICIs are also currently being evaluated as neoadjuvant therapy. However, most results published to date are from early phase trials.

Two phase II trials investigated neoadjuvant ICI administration as monotherapy. The ABACUS trial aimed to investigate the efficacy of two cycles of atezolizumab before cystectomy in 95 patients with MIBC, including 39 (41%) patients ineligible for cisplatin (GFR ≤ 60 mL/min), 70 (74%) with cT2 tumours, and 25 (26%) with cT3-T4 tumours [52]. The CPR rates were 31% in the overall population and 17% in patients with T3-T4 tumours. The final analysis of the study reported 2-year DFS and OS rates of 68% and 77%, respectively. The PURE-01 trial was a phase II trial that evaluated the administration of three cycles of pembrolizumab before surgery [53]. The vast majority of patients were eligible for cisplatin (94%), and 21 patients (42%) had cT2N0 tumours, 27 (54%) had cT3N0, and 2 (4%) had cT3N1 tumours. The trial reported a CPR rate of 42% in the overall population. A follow-up update reported 3-year DFS and OS rates of 74.4% and 83.8%, respectively

An exploratory analysis of ABACUS trial data showed that highly CD8+ T cell-infiltrated tumours and the presence of a cytotoxic T cell transcriptional signature (tGE8) were associated with responses, whereas PD-L1 status and tumour mutational burden were not [54]. In contrast, PD-L1 status with CPS ≥ 10 was correlated with higher CPR in the PURE-01 trial.

The combination of an ICI with chemotherapy has also been evaluated in phase II trials. The administration of four cycles of pembrolizumab + gemcitabine and a split dose of cisplatin in 39 patients with cT2-T4a/N0 tumours led to a CPR rate of 36% and a non-muscle-invasive downstaging rate of 56% [55]. Four cycles of neoadjuvant atezolizumab and cisplatin + gemcitabine administered in the same patient population resulted in a CPR of 41% and a non-muscle-invasive downstaging rate of 69% [56].

Another phase II study evaluated the administration of nivolumab plus cisplatin + gemcitabine in the same population, applying a bladder-sparing strategy [57]. Clinical restaging with magnetic resonance imaging, cystoscopy with biopsy, and urine cytology were pre-planned after the completion of four cycles. Patients with a complete clinical response (cCR) were offered the option to undergo cystectomy or to receive eight further injections of nivolumab followed by surveillance. Among these patients, 33/76 (43%) achieved cCR and 32 of these elected to forgo cystectomy. After a median follow-up of 30 months, eight of these thirty-two patients had undergone cystectomy for later recurrence, and two had experienced metastatic progression, including one who underwent delayed cystectomy.

These promising results regarding the pathologic downstaging and CPR rates achieved with ICIs still require confirmation. Several ongoing randomised phase III trials (KEYNOTE-866, ENERGIZE, NIAGARA) are testing the benefit of adding pembrolizumab, nivolumab, or durvalumab to cisplatin-based chemotherapy in the neoadjuvant setting, followed by an adjuvant ICI [58,59,60]. The PIVOT IO 009 trial is also comparing the efficacy of nivolumab as neoadjuvant followed by adjuvant, either as monotherapy or in combination with bempegaldesleukin, an IL2 pro-stimulant, versus standard of care for cisplatin-ineligible patients, to address the needs of this population [61]. Following the release of the EV-302 trial data, the phase III MK-905 trial is evaluating the combination of EV and pembrolizumab in the peri-operative setting [41].

## 6. Evaluation of ICI in Combination with Chemoradiotherapy

For patients with localized or locally advanced urothelial bladder carcinoma who are unfit for or do not want radical cystectomy, radiation therapy sensitized by chemotherapy is a therapeutic option. Several preclinical studies have shown that radiation therapy could enhance immunogenicity by increasing the number of tumour-infiltrative lymphocytes or the antigen presentation [62]. Moreover, the upregulation of PD-L1 observed with radiation therapy provides rationale to combine this modality with ICI [63].

Two ongoing randomized phase III trials are trying to address this issue. The KEYNOTE-992 trial aims to compare chemoradiotherapy with pembrolizumab/placebo every 6 weeks for up to 9 cycles, for T2-T4aN0M0 bladder cancer with ≥50% urothelial histology, with the primary endpoint being bladder-intact event-free survival [64]. On the other hand, the SWOG S1806 (NTC03775265) phase III trail evaluates chemoradiotherapy +/− atezolizumab for stage II/IIIA bladder cancer, with the same primary endpoint than KEYNOTE-992.

## 7. Biomarker Selection

As it was emphasized above with the discussion of the results from phase III trials, the role of PD-L1 expression as a predictive biomarker to identify responders to ICI is inconsistent between trials. As it is discussed by Meeks et al. in a recent review, these discrepancies might be related to the different PD-L1 assays, antibody clones, or cut points used in these studies, resulting in a different prevalence of PD-L1 positive tumours amongst studies [65].

Other potential predictive biomarkers have been studied. A retrospective study from Graf et al. reported that cisplatin-unfit patients with a tumour mutational burden (TMB) ≥ 10 mut/Mb seem to have better survival outcomes with first line single agent ICI than carboplatin [66]. In addition, a post hoc analysis from the IMvigor210 trial showed that a high Immunotherapy Response Score (IRS) (a weighted score taking into account the TMB, both PD-L1 and PD-1 expression, and the expression of TOP2A and ADAM12) was correlated with OS benefit in both univariate and multivariate analysis under ICI treatment [67]. Interestingly, another recent study established four genetic subtypes of urothelial carcinoma, using different features such as nonsynonymous TMB status, tumour cell purity, ARID1A-mutation, intra-tumoural heterogeneity, and the ratio of non-synonymous to synonymous mutations. Notably, ARID1A mutations and high nonsynonymous TMB were strongly associated with clinical benefit from ICI treatment [68].

Given these data, further trials evaluating ICI should ideally use homogeneous and reproducible criteria and method to identify potential biomarkers of response, such as the PD-L1 expression. Scores and classifications resulting from retrospective studies should also be validated in prospective clinical trials, for instance, by using preplanned patient stratification based on PD-L1, TMB, or ARID1A mutational status.

## 8. Conclusions

**Figure 1:**
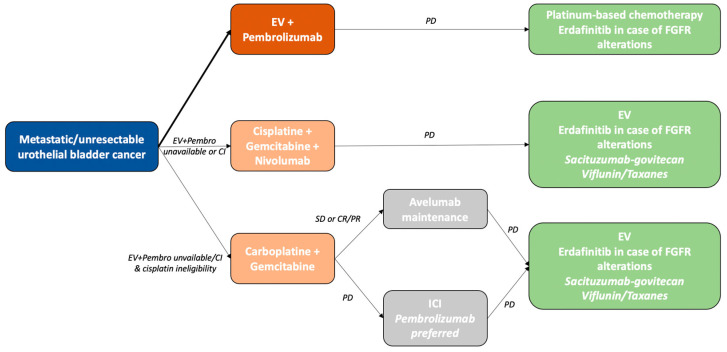
Treatment algorithm for metastatic urothelial bladder cancer, adapted from ESMO guidelines 2024. Treatment choices also depend on the availability of molecules in different countries. CI: contraindicated; PD: progressive disease; SD: stable disease; CR/PR: complete response/partial response.

Although evaluations of immunotherapy in metastatic bladder cancer have long produced contradictory results, the recent positivity of both the Checkmate 901 and EV-302 trials have completely redefined the first-line paradigm for treatment of this disease, as described in the actualised 2024 ESMO guidelines (Figure 1) [69]. The superiority of combined EV plus pembrolizumab over platin-based chemotherapy, including dramatic OS improvement, has led to consideration of this new combination as the first-line standard of care, irrespective of platinum eligibility. Pending approval by the relevant authorities, patients who are ineligible for EV plus pembrolizumab should receive cisplatin plus gemcitabine associated with nivolumab, and cisplatin-ineligible patients should receive carboplatin plus gemcitabine followed by the maintenance of avelumab.

Regarding non-metastatic MIBC, adjuvant nivolumab should be reserved for patients at high risk of recurrence after radical cystectomy, who are not eligible for, or who declined, adjuvant cisplatin-based chemotherapy. Following encouraging trends from phase II studies, data from phase III trials are awaited to precisely identify the efficacy of ICI, alone or in combination with chemotherapy or ADC, in the neoadjuvant setting followed by adjuvant administration. Results of the PIVOT IO 009 trial are also awaited to determine the place of neoadjuvant nivolumab for cisplatin-ineligible patients.
